# Relationship of Surface and Bulk Resistivity in the Case of Mechanically Damaged Fibre Reinforced Red Ceramic Waste Aggregate Concrete

**DOI:** 10.3390/ma13235501

**Published:** 2020-12-02

**Authors:** Marie Horňáková, Petr Lehner

**Affiliations:** Department of Structural Mechanics, Faculty of Civil Engineering, VSB-Technical University of Ostrava, 708 33 Ostrava-Poruba, Czech Republic; petr.lehner@vsb.cz

**Keywords:** concrete, diffusion, durability, red ceramic waste aggregate, resistivity, fibre, mechanical damage

## Abstract

Electrical resistivity is an important physical property of concrete, directly related to the chloride-induced corrosion process. This paper analyses the surface resistivity (SR) and bulk resistivity (BR) of structural lightweight waste aggregate concrete (SLWAC). The studied concrete mixture contained waste material—red ceramics fine aggregate and artificial expanded clay coarse aggregate. Red ceramic is a frequent waste material remaining after the demolition of buildings or unsatisfactory building material production and is among the least used construction waste. Therefore, its use is desirable in terms of sustainability; in some cases, it can reliably replace the conventional aggregate in a concrete mixture. The relationship between SR and BR was determined in the case of standard specimens and mechanically damaged specimens (to 50% and 100% of ultimate strength capacity—USC). Two different instruments were utilised for the investigation—a 4-point Wenner probe meter and an RCON tester. The results of standard specimens support the theoretically derived correction ratio, but in the case of mechanically damaged specimens, the ratio is more scattered, which is related to the mechanical damage and the amount of fibre.

## 1. Introduction

The durability of concrete is defined as its ability to resist a harsh environment (weathering action, chemical attack, abrasion, or any other deterioration process) while retaining its original form, quality, and serviceability [1]. The concrete structure durability is directly linked to a design of sustainable construction, which can also involve the reuse of the waste material. Therefore, to specify the knowledge about usage of waste material is of high interest by many research groups because it is generally known that the amount of waste increases while the natural resources of commonly used aggregates are dwindling [2,3,4].

In this case, the red ceramic waste aggregate was used as a fine fraction of a mixture. It remained after the crushing and grinding of airbricks, which were low quality and could not be sold to customers. The original product was unused, and therefore, the waste aggregate was clean, without any remains of mortar. The studied structural lightweight waste aggregate concrete (SLWAC) designed and tested in [5] consisted of the waste red ceramic fine aggregate (WRCFA), expanded clay coarse aggregate (ECCA), Portland cement, and copper coated crimped steel fibre (CCCSF). Fibre was added to improve the mechanical characteristics, mostly the flexural strength. The influence of crimped steel on the properties of concrete has been studied, e.g., in [5,6]. The mixture was designed to experimentally test the effect of the fine red ceramic aggregate on mechanical characteristics. The used aggregates were pre-soaked, and it was also investigated whether it is possible to design a mixture using the water trapper only in the aggregate. Afterward, the research about SLWAC durability related to chloride ingress was conducted [7]. The diffusion coefficient of chloride penetration was determined by the surface electrical resistivity measurements and the immersion method NT Build 443 [8]. The evaluation of the mechanical and chloride related durability characteristics of the mixture without reinforcement was the aim of the article [7]; the properties of SLWAC were also compared to the ordinary concrete mixture to observe its quality in terms of chloride diffusion.

The effect of mechanical loading on the durability of the concrete has been a subject of many research groups, e.g., [9,10,11,12,13,14,15,16,17,18,19,20]. The relationship between damage and transport properties of concrete has been investigated for more than two decades. In one study [10], an experiment was performed on the chloride migration into the ordinary concrete, which was damaged by different levels of compression and the chloride diffusion coefficient of unloaded concrete was determined by NT Build 492 [21]. The chloride diffusion coefficient is a crucial material parameter for the modelling of realistic behaviour and computation of the service life of concrete structures exposed to chloride penetration. The diffusion of chloride ions into concrete may be influenced by several factors (concrete material properties, temperature, age, moisture content and environmental conditions), which were investigated in the undamaged concrete [22,23,24,25,26,27]. It was proven that chloride transport properties are directly connected to the porosity of undamaged concrete. When the concrete is damaged by the applied load and subjected to a chloride environment, the chloride ingress into concrete is a rapid process, which is significantly influenced by the extent of the damages in the concrete. This process is analogous to the fact that the permeability of damaged concrete increases by several orders of magnitude, which was described and proven in [28]. The effect of mechanical load on the diffusivity of concrete was investigated in the reinforced concrete beam subjected to flexural load in research [11], and similar studies were conducted in [12,13,14].

In the case of modelling of the construction’s service life and assessing the bearing capacity of the concrete structure, it is also necessary to consider the cracking and changes in the pore structure of the concrete. Some research has presented the models for analysing the simultaneous effects of mechanical and environmental loads of concrete structures [15]. One of the main inputs into the model is a diffusion coefficient, which should involve the effect of mechanical damage [16,17]. Therefore, finding a sufficient methodology to determine the diffusion coefficient of damaged concrete is necessary to ensure proper modelling and prediction of the service life.

The diffusion coefficient can be determined by many methods, e.g., immersion methods [8,29,30], rapid chloride permeability test [21,31,32], or resistivity techniques [33,34]. NT Build 443 [8] is recommended as a reference test method for chloride penetration into concrete [35], but its highly labour- and time-demanding process of testing has led to the development of more effective procedures, such as the electrical resistivity measurements [36].

Electrical resistivity can be measured on the surface of the concrete (SR, [33]), or as the bulk electrical resistivity (BR, [34]) on a concrete core. The results of bulk resistivity should be more precise, but in practice, it is not always possible to drill a core out of the structure for testing of the bulk resistivity; therefore, the measurement of surface electrical resistivity comes in handy. However, the accuracy of surface resistivity may be influenced by the amount of pressure on the concrete and the location of the measurements on the surface.

In this study, one of the main aims was the identification of the relationship between the addition of fibre to the SLWAC and its consequent change in the electrical resistivity obtained by the two methods (surface and bulk electrical resistivity) on the standard cylindrical specimens. A correlation may be found between these two methods, which may depend on the composition and volume of fibre. The determined correction ratio may be used to convert SR to BR for the calculation of the diffusion coefficient. Afterwards, mechanically damaged specimens were tested to determine the electrical resistance of the loaded concrete. The influence of partial and full loading on the electrical resistance of SLWAC was investigated. Two non-destructive testing instruments were used in this project to measure the electrical resistivity of concrete to ensure the accuracy of the data; the four-point Wenner Probe was used to measure SR according to [33], and the RCON tester [37] was utilised according to [34] to measure BR at a wide range of frequencies to assess the influence on the results of BR. Although measuring the bulk electrical resistivity by RCON is considered non-destructive, one should note that, in practice, the core needs to be drilled out of the structure.

Based on the information mentioned above, the objectives of the article have been set as:

- Evaluation of the application and the precision of two advanced non-destructive tests (NDT) on the new types of specimens and comparison to the standard cylindrical specimens;
- Investigation of SLWAC electrical properties related to a different amount of steel fibre;
- Investigation of SLWAC electrical properties of differently loaded samples;
- Investigation of SLWAC electrical properties in the combination of those two groups (different amount of fibre and different mechanical damage);
- Evaluation of the experimental correlation of the bulk and surface resistivity of concrete on all types of the SLWAC mixtures.

## 2. Materials and Preparation of Specimens

### 2.1. Used Materials and Mixture Composition

**Figure 1:**
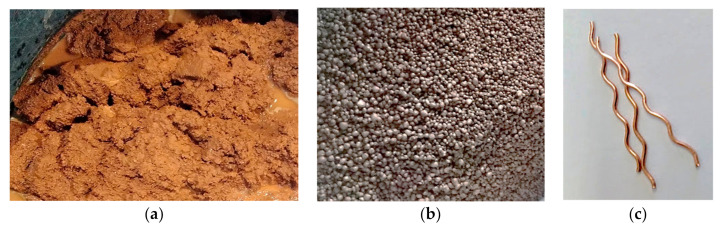
Waste red ceramic fine aggregate (**a**); expanded clay coarse aggregate (**b**); copper coated crimped steel fibre (**c**).

The waste red ceramic fine aggregate (WRCFA, Figure 1a), with a loose density of 1183.8 kg∙m^−3^ and absorptivity of 46%, and expanded clay coarse aggregate (ECCA, Figure 1b), with a loose density of 318.8 kg∙m^−3^ and absorptivity of 36%, were used for creating the mixture. These aggregates were pre-soaked, and no additional water was used in the mixture. Another component of the mixture was Portland Cement I 42.5 (Warta cementownia, Trebaczew, Poland). As a reinforcement, the copper coated crimped steel fibre (CCCSF, Figure 1c) was added in various percentages of the volume of the mixture. The amounts of specific components are given in Table 1. The process of selecting the material, design and testing of the aggregates, fibre, and resulting mechanical characteristics of the mixture are described in detail in [5]; therefore, it is not further presented in this article.

Based on Table 1, one should note that three types of concrete were evaluated, one which does not contain any fibre and two with fibre reinforcements of 1.0% and 1.5%. The volumes of added fibre represent the most common amount of fibre added to concrete, because adding less than 0.5% does not noticeably influence the concrete characteristics, and adding more than 1.5% requires changes of the mixing procedure or adding an admixture to provide good workability.

### 2.2. Characteristics of the Mixture

The concrete was designed to observe the characteristics of a mixture made of fine red ceramic waste aggregate. The composition was created based on the requirements of limited density (1800 kg∙m^−3^), using only the pre-soaked aggregate and no more additional water, limited amount of cement, good workability of the mixture, and closed structure of the specimen. These requirements were fulfilled, the density of concrete was determined to be 1366.63 kg∙m^−3^ for 0% of fibre, 1450.29 kg∙m^−3^ for 1.0% of fibre, and 1484.89 kg∙m^−3^ for 1.5% of fibre. The consistency of concrete was determined according to [38] as S2, for 0% and 1.0%, and S1 for 1.5%. The compressive strength was tested on standard cubes [39], and it was measured as 15.4 MPa for 0% of fibre, 14.7 MPa for 1.0% of fibre, and 17.1 MPa for 1.5% of fibre. Additionally, other characteristics, namely splitting tensile strength, static modulus of elasticity, dynamic modulus of elasticity, flexural characteristics and shear strength, were tested and can be found in [5], but these characteristics are not so important for this research.

### 2.3. Preparation of Specimens

**Figure 2:**
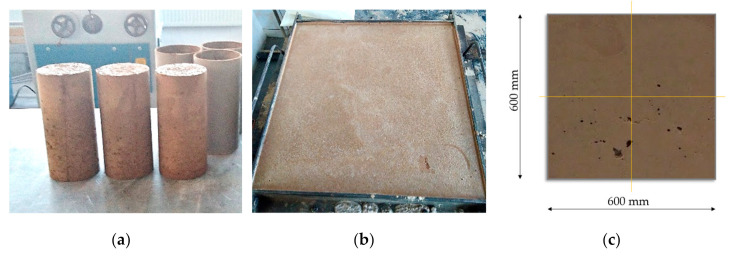
Cylinders (**a**); plate (**b**); how the plate was cut in 4 pieces (**c**).

The specimens in the form of three cylinders of every type of mixture with a diameter of approximately 100 mm and a height of 220 mm, and one plate of every kind of mixture with dimensions of approximately 90 × 600 × 600 mm were cast (see Figure 2), and after unforming they matured in water for 28 days. Afterward, the cylinders were kept in water for the whole time, but the plates were cut into 4 pieces with dimensions of 90 × 300 × 300 mm and dried in a dryer for three days, because the intention was to test their thermal characteristics in a machine that requires these dimensions and dried specimens. The specimens were prepared and loaded in laboratories of Koszalin, University of Technology and cut and tested in the laboratories of VSB—Technical University of Ostrava—as a part of projects SGS SP2019/120 and SGS SP2020/126. Before the resistivity testing, the plates were also saturated.

### 2.4. Loading and Cutting of Samples

**Figure 3:**
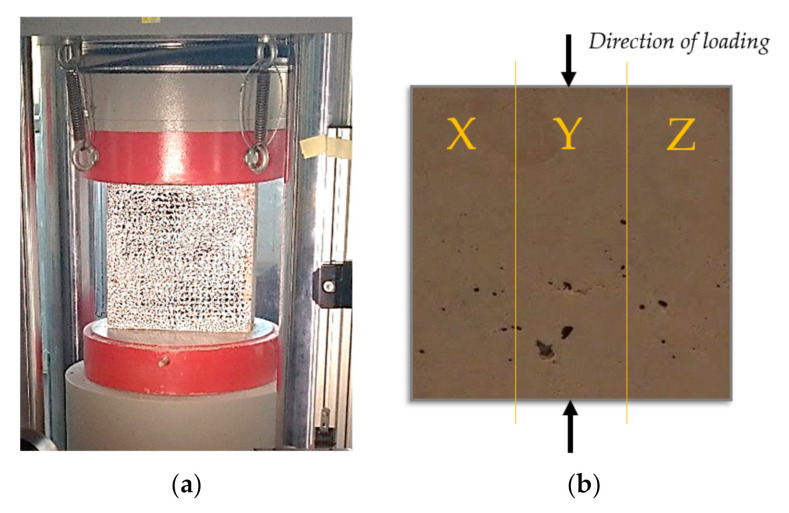
Loading of the plate (**a**); how the loaded plate was cut into 3 pieces (**b**).

The plates were mechanically damaged 120 days after casting, with loadings of 0%, 50% and 100% of their ultimate strength capacity (USC). The reason for the black and white staining of the plates in Figure 3a is for the use of a method based on optical measurements of strains with the digital image correlation system [40]; however, the resulting strains of the plates were not used in terms of electrical resistivity testing, and therefore are not listed in this article.

The loading of the sample is shown in Figure 3a. After the loading, the specimens were cut vertically into three pieces, as shown in Figure 3b. The reason for this last cut is the limited dimensions of the sample for the bulk resistivity measurements. After the final cutting, all of the samples were measured, and the dimensions are given in Table 2, where *L* is the length, *w* is the width, and *h* is height of the specimen. The resistivity measurements were conducted after almost a year after the loading; therefore, the specimens were definitely matured when they were tested. The dimensions of cylinders are given in Table 2 as well, and *L* is the length and *d* is the diameter of the cylinder.

## 3. Methodology of Resistivity Measurements

Electrical resistivity is a characteristic of a concrete’s ability to withstand the passage of an electric current. It can be used for various types of problems—to identify early age characteristics of fresh concrete, to determine the moisture content and the connectivity of the micropores in the concrete, to detect and monitor the cracks in concrete, etc. [41]. Moreover, the specific resistivity is an indirect measuring method of the degree of chloride ion diffusion. The factors that may affect the concrete electrical resistivity are w/c ratio, aging, pore structure and specimen geometry, moisture content, temperature, electrode spacing and presence of reinforcement. There are several terms related to the resistivity methods, namely resistance and conductivity, which may cause confusion. Conductivity represents the conductivity of concrete and the inverse equivalent of resistance. Resistance is theoretically defined similarly to resistivity, but resistivity is normalised to unit cross-section and length [42].

As has been mentioned above, the chloride diffusion coefficient can be indirectly determined by the resistivity measurements by the Nernst–Einstein equation—Equation (1). The procedure of the measurement and calculation process has been described in several papers [23,24,43].

(1) $$D=\frac{R\cdot T}{Z^{2}\cdot F^{2}}\cdot \frac{t_{i}}{\gamma_{i}\cdot C_{i}\cdot \rho_{BR}}$$

where *D* is the diffusivity of the chloride ion (m^2^·s^−1^); *R* is the universal gas constant (J/K·mol); *T* is the absolute temperature (K); *Z* is the ionic valence (-); *F* is the Faraday constant (C/mol); *t_i_* is the transfer number of chloride ion (-); *γ_i_* is the activity coefficient for chloride ion (-); *C_i_* (C/mol) is the concentration of ions *i* in the pore water, and *ρ_BR_* is the bulk electrical resistivity (Ω·m).

### 3.1. Surface Resistivity

**Figure 4:**
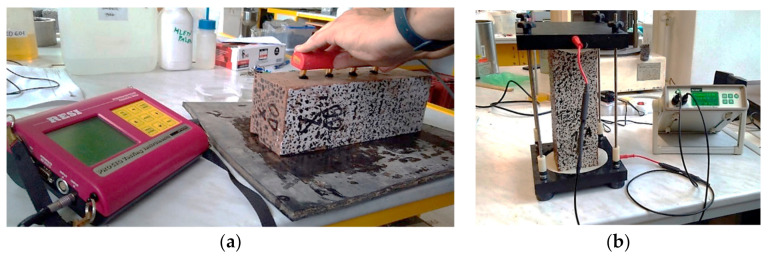
Testing of the surface resistivity (SR) with the Wenner probe (**a**); bulk resistivity (BR) with RCON (**b**).

The Wenner probe (Proceq, Schwerzenbach, Switzerland), used for measuring of the SR (see Figure 4a), consists of four electrodes with a spacing of approx. 50 mm. External electrodes applied electric current, and the internal electrodes measure the difference in electric potential [33]. In standard procedures, the method is used on continuously saturated concrete cylindrical samples; in this study, the procedure was also undertaken for concrete plate specimens, which were dried and then saturated. The surface resistance was measured on all four longer sides, after which the mean and standard deviation were evaluated.

### 3.2. Bulk Resistivity

RCON is a non-destructive testing tool for measurment of the BR [37] (see Figure 4b). The impedance Z (Ω) was calculated from the measured values of voltage and applied current. The impedance phase can be calculated by determining the difference between the voltage and current phases. During the experiment, the impedance phase was obtained at almost zero, which means that the measured impedance was in fact the resistance of the concrete.

### 3.3. Correlation Between the Surface and Bulk Resistivity

It was derived in [44] that the ratio of two different resistivity types is equal to 2.63 for the cylinder with a diameter of 100 mm, length of 200 mm and probe spacing of 50 mm. The theoretical ratio of surface and bulk resistance was calculated in [45] as 0.33. The correlation between bulk and surface resistivity data has been studied by many research groups, e.g., [25,27,46] and it was observed that in the case of different groups of binary and ternary mixtures, the coefficient of determination (R^2^) for linear trend line was higher than 0.8 and sometimes close to 1.

In this project, the correlation between surface and bulk resistivity of undamaged SLWAC without reinforcement was determined based on the undamaged standard cylindrical specimens. Then, the influence of the amount of fibre on SR and BR was obtained. The last correlation related to the effect of mechanically damaged concrete with and without reinforcement was studied related to the two methods of measuring the electrical resistivity. Based on the research [44], the theoretical ratio of two different types of resistivity for the analysed cylinders was calculated as 2.53 and this value was also used for the evaluation in this paper. New ratios for the reinforced and damaged specimens are determined in the next section.

## 4. Results and Discussion

### 4.1. Results of Surface Resistivity

**Figure 5:**
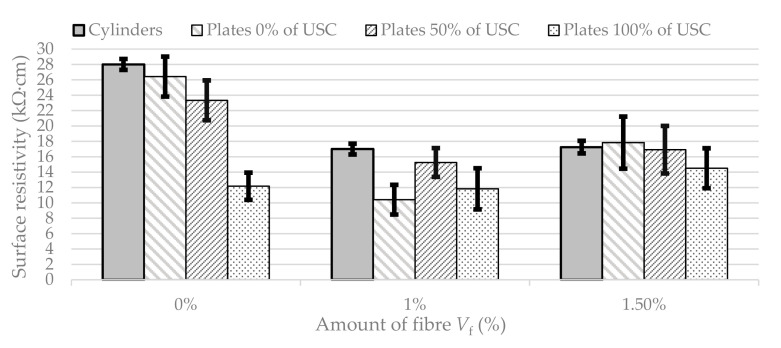
Surface resistivity of standard cylinders and plates with different amount of fibre (*V_f_*) and values of load (USC).

For evaluation of the results, the standard cylinder specimens were considered as the reference samples. The results of the surface resistivity of cylinders and plates can be seen in Figure 5. The results of measured surface resistivity in the form of mean value and standard deviation for all samples are given in Table 3. The variance of the measured values displayed through the standard deviation was two to three times smaller for the cylinders than for the plates. This is likely caused by the geometry of electric wave propagation and is reflected in both surface and bulk resistivity measurements.

It can be observed that SR in the case of standard cylinders decreases with the amount of fibre (39% difference for 1.0% of fibre and 43% difference for 1.5% of fibre in comparison to 0% of fibre), which can be caused by disruption of structure homogeneity or even the corrosion of the fibre.

In plates with 0.0% of loading, the resistivity also decreases. It is interesting that it is not a gradual decline, because the specimens with 1.5% of fibre report 28% higher resistivity than specimens with 1.0% of fibre and 35% lower than specimens without reinforcement. The reason could be the pores in the structure formed by the higher amount of fibre. A similar trend can be seen also in the 50% loaded plates; SR in the case of 1.0% of fibre is 35% lower than 0.0% and 1.5% is only 28% lower than 0% of fibre. The plates loaded to 100% even have an opposite trend; the resistivity of specimens without reinforcement is about 3% higher than specimens with 1% of fibre, but it is 20% lower than in the case of 1.5% of fibre, which indicates that when the SLWAC is more reinforced, the surface resistivity in the case of full mechanical damage even increases.

### 4.2. Results of Bulk Resistivity

**Figure 6:**
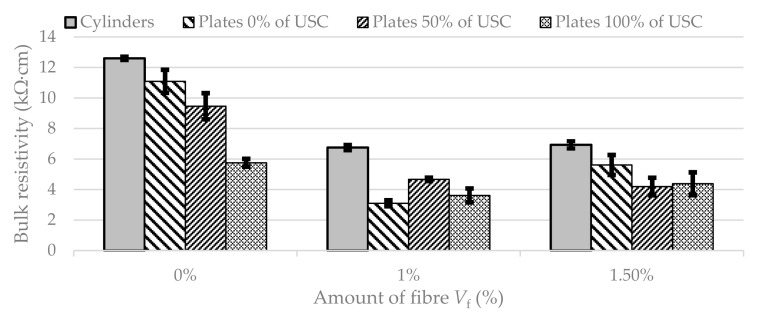
Bulk resistivity of standard cylinders and plates with different amount of fibre (*V_f_*) and values of load (USC).

The same approach for the evaluation of bulk resistivity results was used and the reference standard cylinders and plates were tested. The results can be seen in Figure 6. It can be observed that BR in standard cylinders decreases about 46% for 1% of fibre and then slightly increases (45% in comparison to 0.0% of fibre) for 1.5% of fibre. The results of bulk resistivity in the form of mean value and standard deviation are given in Table 4.

In the case of plates with zero and full loading, the resistivity has the same trend as the standard specimens; specimens with 1.0% of fibre report a reduction in BR as: 30% for zero loading and 20% for full loading in comparison to unreinforced plate specimens. Specimens with 1.5% of fibre report a reduction in 20% in the case of zero loading and 11% in the case of full loading in comparison to unreinforced plate specimens. Gradual decline dependent on the amount of fibre can be seen in the case of 50% of loading (about 20% for 1.0% of fibre and 23% for 1.5% of fibre).

### 4.3. Correction Ratio and Correlation of Results

The correction ratio was determined by a simple division of surface and volume resistivity [44]. The ratios for all three concrete mixtures in the case of standard cylindrical specimens are given in Table 5.

It can be seen, that results of cylinder specimens are close to Morris’s 2.53 and the difference is approximately 7% on average. One should note that the ratio increases for 1% of fibre in comparison to 0% of fibre and then slightly decreases for 1.5% of fibre in comparison to 1% of fibre. Of course, it is necessary to take the results with caution because the measurement of surface resistivity has a relatively high inaccuracy, as has been proven, e.g., in [23]. While considering specific tolerances, it is possible to declare these results as sufficient.

More interesting are the resulting correlations between surface and volume resistivity in plate specimens, where there are different values of the amount of fibre and specimens also partially and fully loaded. These correlations are also listed in Table 5. In unreinforced plate specimens, the ratio is similar to Morris’s even when the specimens were loaded. For 1.0% of fibre, the ratio is about 30% higher than Morris’s as well, even in the case of loaded specimens, and it seems it is not highly related to the loading. Plates with 1.5% of fibre report an increasing in the ratio by about 39% on average in comparison to Morris’s, but the ratio is lower in the case of zero loading compared to 1.0% of fibre but higher than 0.0% of fibre. In the case of half loading, the ratio increased gradually by about 23% compared to 1.0% of fibre. For the full loading, the ratio increased only about 1% in comparison to 1.0% of fibre.

**Figure 7:**
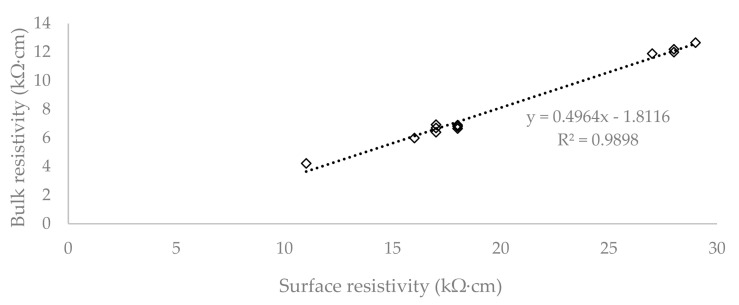
Correlation of surface and bulk resistivity on the cylinder specimens with coefficient of determination.

**Figure 8:**
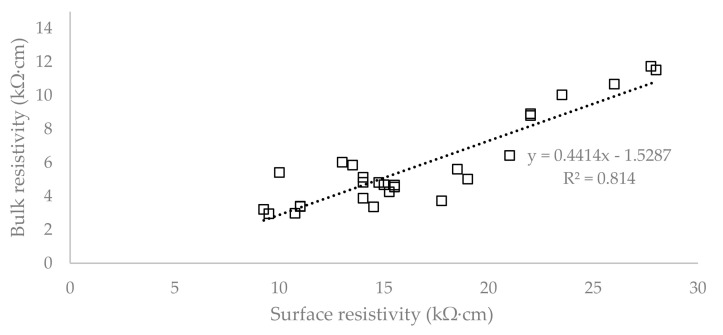
Correlation of surface and bulk resistivity on the plate specimens with coefficient of determination.

**Figure 9:**
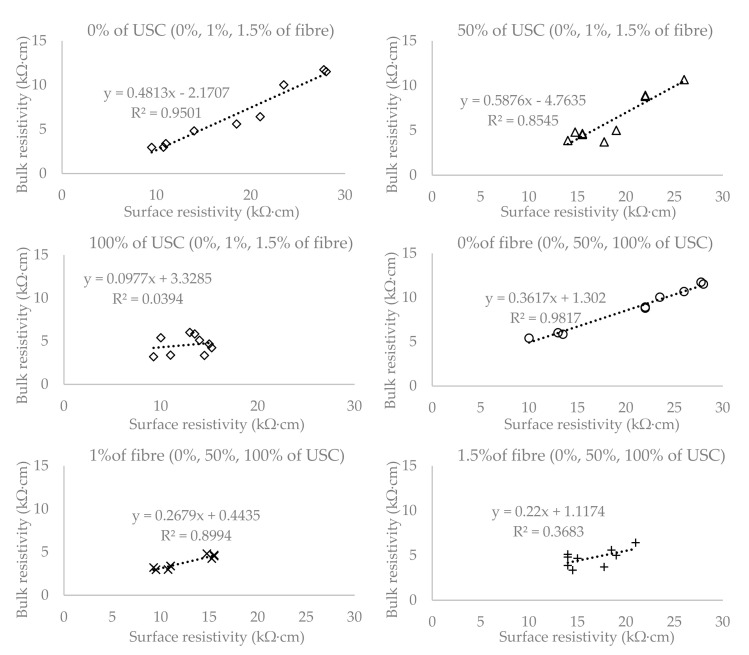
Correlation of surface and bulk resistivity on the plate specimens with coefficient of determination on different groups according to the loading and the amount fibre.

To evaluate the correlation between SR and BR, the coefficient of determination (R^2^) was chosen, which is shown for all the cases in Figure 7, Figure 8 and Figure 9.

Figure 7 shows that the cylindrical specimens report a coefficient of determination equal to 0.9898, which demonstrates a high degree of correlation between the SR and BR tests. The plate specimens report a coefficient of determination equal to 0.814 and it can be seen in Figure 8 that there are large deviations from the linear regression. The degree of correlation agreement in the case of separate groups can be observed in Figure 9. Its value is much smaller in the group with the plates loaded to 100% (0.0394) and the group with concrete reinforced by 1.5% of fibre (0.3683). The reason for such a low coefficient of determination could be the fact that the surface resistivity is affected by the cracks and displacement of the individual components of concrete and reinforcement, while in the case of bulk resistivity, the electric current passes through the entire volume of the specimen, and the cracks on the surface do not affect the results. The other groups show a coefficient of determination over 0.85.

## 5. Conclusions

1. Measuring the surface resistivity is indisputably very advantageous because the construction does not need to be destroyed for the test. However, it is necessary to consider the correction ratio between surface and bulk resistivity, namely for determining the diffusion coefficient, where the bulk resistivity is an essential input into the calculation. The determination of the correction ratio should be of high interest in the case of other concrete mixtures tested by the surface resistivity method to predict their durability.
2. In this study, the correction ratio and correlation in terms of the coefficient of determination of bulk and surface resistivity of the SLWAC were investigated, finding:
  (a) The correction ratio of standard cylinder samples is in proper agreement with the previous research study regardless of the volume of fibre.
  (b) Correction ratios of plates with the different values of preliminary load and different amount of fibre have more significant scattering (between 2.12 and 4.03), and it is related to the load damage and the amount of fibre.
  (c) The coefficient of determination of cylindrical samples between SR and BR is 0.9898. In the case of the plates, the coefficient of determination is 0.814 without the consideration of amount of fibre and the load value.
  (d) Both bulk and surface electrical resistivity are well correlated for standard cylinders, for non-loaded or 50% loaded plates samples, also for plates with 0% and 1% of fibre content, as evidenced by the high value of the coefficient of determination.
  (e) Conversely, for the plates containing 1.5% of fibre and plates loaded to 100%, the coefficient of determination is very low and shows a very low correlation. These conclusions deserve further research also in the case of other reinforced waste aggregate mixtures.
3. It can be observed that the resistivity is also correlated to the compressive strength. Based on results found in [5], the compressive strength was lower in the case of 1.0% of fibre and the same tendency can be seen in the case of resistivity. It would be desirable to evaluate the influence of compressive strength to resistivity in the case of SLWAC and also other waste concrete mixtures.

## Figures and Tables

**Table 1:** Mixture proportions of a cubic meter of mixture [5].

Composition	Quantity (kg∙m^−3^)	Absorbed Water (kg∙m^−3^)
WRCFA-dry	378.38	322.33
ECCA-dry	247.07	138.98
Cement	320.49	-
CCCSF, *V*_f_ = 0.0%	0.0	-
CCCSF, *V*_f_ = 1.0%	78.0	-
CCCSF, *V*_f_ = 1.5%	117.0	-

**Table 2:** Dimensions of the cylindrical and plate specimens.

Amount of Fibre		0% of USC			50% of USC			100% of USC			Cylinders	
*V*_f_	-	*L* (mm)	*w* (mm)	*h* (mm)	*L* (mm)	*w* (mm)	*h* (mm)	*L* (mm)	*w* (mm)	*h* (mm)	*L* (mm)	*d* (mm)
0.0%	X	297	100	87	301	99	90	296	99	89	223	104
	Y	297	99	88	299	99	90	297	99	89	222	103
	Z	297	89	89	299	89	89	297	99	89	222	104
1.0%	X	294	99	95	296	99	94	296	99	93	224	102
	Y	295	99	94	296	99	94	298	99	94	221	103
	Z	296	95	90	297	93	90	297	94	90	223	104
1.5%	X	298	96	89	295	98	88	295	101	88	222	104
	Y	297	96	89	296	99	88	296	94	88	222	103
	Z	297	93	82	296	88	88	296	91	88	223	103

**Table 3:** Results of surface resistivity of structural lightweight waste aggregate concrete (SLWAC).

-	Cylinders		Plates					
-	Mean	SD	Mean (kΩ·cm)			SD (kΩ·cm)		
*V*_f_	(kΩ·cm)	(kΩ·cm)	0% of USC	50% of USC	100% of USC	0% of USC	50% of USC	100% of USC
0.0%	28.00	0.71	26.41	23.33	12.16	2.59	2.59	1.77
1.0%	17.00	0.70	10.41	15.25	11.83	1.93	1.87	2.67
1.5%	17.25	0.82	17.83	16.91	14.50	3.38	3.09	2.59

**Table 4:** Results of bulk resistivity of SLWAC in the form of mean value and standard deviation.

-	Cylinders		Plates					
	Mean	SD	Mean (kΩ·cm)			SD (kΩ·cm)		
*V*_f_	(kΩ·cm)	(kΩ·cm)	0% of USC	50% of USC	100% of USC	0% of USC	50% of USC	100% of USC
0.0%	12.60	0.12	11.09	9.45	5.75	0.75	0.85	0.25
1.0%	6.75	0.18	3.09	4.66	3.61	0.19	0.11	0.45
1.5%	6.93	0.24	5.61	4.19	4.38	0.65	0.57	0.74

**Table 5:** Mean correction ratio of surface and bulk resistivity.

Amount of Fibre	Cylinders Ratio (-)	Plates Ratio (-)		
*V*_f_	-	0% of USC	50% of USC	100% of USC
0.0%	2.21	2.38	2.47	2.12
1.0%	2.56	3.36	3.27	3.27
1.5%	2.31	3.18	4.03	3.31
